# Epidemiology of Schistosomiasis and Usefulness of Indirect Diagnostic Tests in School-Age Children in Cubal, Central Angola

**DOI:** 10.1371/journal.pntd.0004055

**Published:** 2015-10-16

**Authors:** Cristina Bocanegra, Sara Gallego, Jacobo Mendioroz, Milagros Moreno, Elena Sulleiro, Fernando Salvador, Nicolau Sikaleta, Arlette Nindia, Daniel Tchipita, Morais Joromba, Sebastiao Kavaya, Adrián Sánchez Montalvá, Teresa López, Israel Molina

**Affiliations:** 1 Special Program for Infectious Diseases Drassanes-Vall d’Hebron. PROSICS Barcelona, Spain/Hospital Nossa Senhora da Paz, Cubal, Angola; 2 Hospital Nossa Senhora da Paz, Cubal, Angola; 3 EpidemiologyDepartment. University Hospital Vall d’Hebron, Barcelona, Spain; 4 Microbiology Department. University Hospital Vall d’Hebron. PROSICS Barcelona, Spain; 5 Infectious Diseases Department. University Hospital Vall d’Hebron. PROSICS Barcelona, Spain; University of Florida, UNITED STATES

## Abstract

**Introduction:**

Schistosomiasis remains a public health major problem and little is known in many areas, mainly in Sub-Saharan Africa

**Objectives:**

To assess the burden and risk factors of schistosomiasis and intestinal parasitic helminthes in the children of Cubal, Angola, and to compare different diagnostic approaches for urinary schistosomiasis under field conditions.

**Methods:**

A cross-sectional study was conducted. Urine and faeces samples of school children were microscopically studied. A random sample of children was obtained from an alphabetically arranged list of children, taking one of two children. Urine dipstick, colorimetric test and macrohaematuria were considered as indirect diagnostic methods and compared to direct urine examination. Possible risk factors for the infection were sex, age, distance to the river and previous treatment with praziquantel; the assessment was performed using Chi-square test.

**Results:**

A total of 785 (61.18%) children showed *S*. *haematobium* eggs in urine; children living within 500 meters from the river had a higher odds for infection: Odds ratio 1.97 (1.45–2.7 CI 95%); urine dipstick showed sensitivity of 96% and specificity of 61.3%, with a positive predictive value; colorimetric test showed sensitivity of 52.5%, specificity of 74.6% and a positive predictive value of 77%. Proteinuria was present in 653 (51.1%) children, being more frequent in children with *S*. *haematobium* in urine (75.2%); 32 of 191 stool samples (16%) showed the presence of other intestinal parasites and 8 (4%) for *S*. *haematobium*.

**Conclusions:**

Prevalence of urinary schistosomiasis in our study area is much higher than the national average, considering it as a high-risk community. Proximity to a source of water was a risk factor for the infection. Indirect tests, as urine dipstick and colorimetric test, were useful tools for diagnosis, due to ease of use and low cost. Proteinuria was a common finding, probably showing an early structural damage due to schistosomiasis in this group of children.

## Author Summary

Schistosomiasis is a parasitic disease that affects both the individual and the community as a whole. Its transmission and life cycle depend on non-safe water, as infection is acquired bathing in fresh water. As in many neglected diseases, information about it is scarce in many areas, mainly in Sub-Saharan Africa. It is the case in Angola, where a prolonged civil war has hampered development and research for many years. This paper highlights the epidemiology of the disease in Cubal, main town of a rural area in central Angola. It shows a very high prevalence of urinary schistosomiasis in the school-age population of the area; the risk is higher among the children living closer to one source of water. This population must be considered as a high-risk community for the development of complications. We advocate for the use of indirect tests for diagnosis, as urine dipstick and colorimetric test, due to their easiness of use and low price. At the same time, it relates the presence of the disease with signs of severity that are usually observed at an older age, suggesting that prevention must be initiated as soon as possible. Overall, this paper will provide the research and medical community with essential data for future intervention strategies to manage schistosomiasis in the region.

## Introduction

Human schistosomiasis remains, after malaria, one of the main sources of morbidity and mortality in endemic countries, with major consequences on public health and economy, especially in Sub-Saharan Africa. It is considered as one of the most prevalent parasitic infections in the world with about 200 million people infected and 20 million with a severe form of the disease. In Sub-Saharan Africa, it is estimated that more than 200,000 deaths per year are due to schistosomiasis [1]. Other consequences of the disease, as anemia, chronic pain, growth stunting and nutritional and cognitive impairment have also been recently demonstrated affecting not only the individual, but also the community as a whole, hampering its development and perpetuating poverty [2,3].

In 1984 the WHO Expert Committee for the Control of Schistosomiasis approved a strategy for morbidity control, mainly based on periodic mass treatment in school-aged children, due to the availability of safe and effective drugs, and ease to administer in a single dose [4]. Then, one of the first steps required to eliminate the disease is a study of the local characteristics of the disease, in order to maximize the impact of the public health measures such as mass drug administration of praziquantel, by prioritizing the places with a higher prevalence. Although schistosomiasis may be eradicated in focal areas through current mass drug administration programmes, global control and elimination will require an integrated approach with multifactorial and coordinated interventions. Thus, reliable data on the epidemiology of the disease will be useful for further actions.

However, these data are scarce in Sub-Saharan Africa and especially in Angola, where there has only been a single recent study, performed in the north of the country, which informed of the existence of both intestinal and urinary types of schistosomiasis [5]. A recent national survey was conducted by the Ministry of Health in order to determine the impact of mass drug administration campaigns as per the National Neglected Tropical Diseases guidelines. The estimated overall prevalence of urinary schistosomiasis was 28% [6]. Data regarding urinary schistosomiasis in Cubal district, in Central Angola, are also scarce and come from old studies performed in the 1970s, with an estimated prevalence between 35% and 85% [7]. Concerning intestinal schistosomiasis, there are only isolated reports⁷. Prevalence and intensity of the infection and description of intermediate hosts are unknown. The main aim of this study was to determine the prevalence of urinary and intestinal schistosomiasis and other intestinal parasitic infections in the children of Cubal. Other objectives were to compare different diagnostic approaches under field conditions and the assessment of risk factors related to the infection.

## Methods

A cross-sectional study was conducted between March 2013 and February 2014 in Cubal, the capital city of the district.

### Study population and data collection

The district of Cubal is situated in western-central Angola. It has an estimated population of 322,000 with 151,000 (47%) children under 15 [8]. In a primary field approach to possible schistosomiasis infection sources, we noted a pond in the northeastern area of the city, and the Cubal River, which crosses the city by its southern border. We found no other water facility nearby used by the local population.

Children studying in years 4 and 5, corresponding to 9–10 years old, were recruited in every school in Cubal. In order to be representative, we screened at least 50 children in each class, from a total of approximately 100 children in each class. We randomly selected one of consecutive pairs of children, in alphabetical order. Samples were collected during daily classes.

Participation in the study was voluntary, and with prior parental consent. We excluded children whose parents or legal guardians objected to their participation. Age, sex, school and neighbourhood of each child, as well as previous treatments with albendazole/mebendazole or praziquantel within the two previous months, were recorded. Distance from their neighbourhood to a source of water was estimated on a local actualized map.

### Sample size calculation

Sample size calculation was based on the estimated prevalence recorded in the recent National Survey. For a local prevalence of schistosomiasis of 30% [6], sample size was estimated as 1,280 children under 15 years old for a margin of error of 2.5% (95% confidence interval, [CI 95%]). In order to avoid missing data, finally 1,425 cases were recorded.

### Urine analysis for *Schistosoma haematobium* detection

Urine samples were requested from all children included in the survey. Micro and macrohaematuria were evaluated for the initial detection of schistosomiasis through three indirect tests: a colorimetric test, a urine dipstick test, and the visual examination of urine by the researchers.

In the colorimetric test, a picture with six haematuric and non-haematuric urines was shown to the children to identify what colour corresponded to their daily urine. Urine colours 1, 2 and 3 were considered non-haematuric and urine colours 4, 5 and 6 haematuric.

Microscopic haematuria was detected through urine dipstick test (Combi-Screen 11SYS). The results were expressed with a number of crosses, with one cross denoting 5–10 erythrocytes per field; two crosses denoting 50 erythrocytes per field and three crosses denoting 300 erythrocytes per field. The same dipstick also detected proteinuria (1 cross = 30 mg/dL; 2 crosses = 100 mg/dL; 3 crosses = 300 mg/dL).

Macroscopic haematuria was determined by one trained researcher. Two blinded independent researchers examined with optical microscopy all the urine samples that had micro/macrohaematuria in at least one of the indirect tests.

The definitive diagnosis of urinary schistosomiasis was made if *S*. *haematobium* eggs were present on the sample. To optimize resources, only one of every five urine samples with three negative results in the indirect tests for haematuria was examined.

### Stool diagnostic technique for schistosomiasis and other parasitic infections

Stool samples were requested from one in two of the selected children. Microscopical examination was performed with the formol-ether diagnostic method by the same two blinded researchers.

### Statistical analysis

Continuous variables were expressed as means and standard deviations (SD). The estimated prevalence of schistosomiasis was calculated overall and stratified by age, gender, school and neighbourhood. The normal distribution of quantitative variables was tested through the Shapiro–Wilk test. Differences of normally distributed variables between groups were evaluated with *t*-tests and ANOVA. For the non-normally distributed variables, differences were evaluated with the Mann–Whitney U test. Differences on infection status, and odds ratio were evaluated with Chi-square tests. We considered as possible risk factors of infection sex, age, distance to one of the two possible sources of water (pond and river) and previous treatment with praziquantel. The assessment of factors associated with infection was performed using Chi-square tests.

Sensitivity, specificity, positive predictive value and positive and negative likelihood ratios of the three indirect tests were determined using direct microscopical observation of *S*. *haematobium* eggs as the gold standard technique. Comparison of sensitivity, specificity, positive and negative predictive value of the different diagnostic approaches was made based on magnitude; no specific statistical testing was performed.

Data collection and calculations were performed using EpiInfoTM version 7 and STATA version 11.

### Ethical aspects

The project was approved by the Vall d’Hebron Research Institute Ethics Committee and by the Health and Education regional institutions. We conducted previous informative talks with school principals, teachers, parents, and community leaders; informed consent was obtained from all parents or legal guardians. Every student diagnosed with schistosomiasis or intestinal parasites received appropriate treatment.

## Results

### Characteristics of the study population

A total of 1,425 children from 19 schools were initially included in the study. Participation ranged from 29 to 192 children among the different schools. Due to drop outs during the recruitment process, urine samples were finally obtained from 1,283 children (90%) and stool samples from 191 children (13.4%). A total of 655 (51.05%) children were boys. Mean age was 8.7 years (SD 3.2). Only 65 children (4.5%) reported receiving treatment with praziquantel in the two previous months and 22 (1.5%) with mebendazole or albendazole.

### Prevalence of urinary schistosomiasis

**Fig 1:**
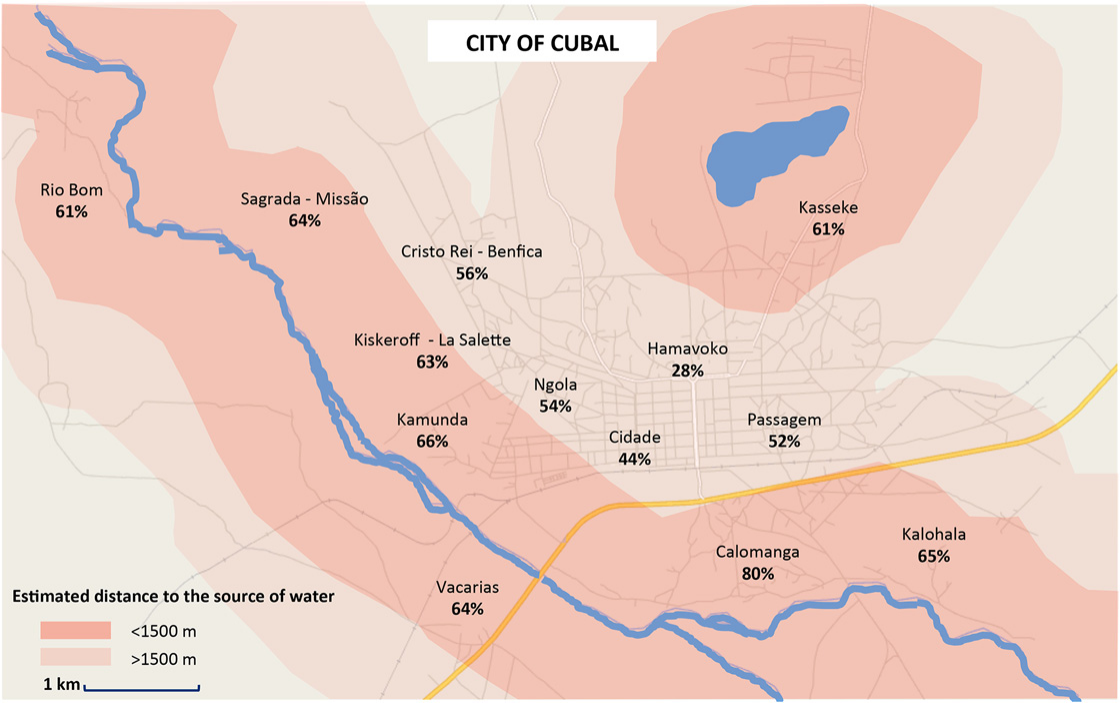
Prevalence of urinary schistosomiasis considering 13 distinct neighborhoods and their relationship to the sources of water.

Seven hundred and eighty five children were considered to be infected by *S*. *haematobium*. A crude assessment of prevalence of schistosomiasis infection in the school age population of Cubal considering only the samples analysed was 61.18% (CI 95%; 58.4–63.9). The prevalence of urinary schistosomiasis in each neighbourhood of the city of Cubal is shown on Fig 1. As *S*.*haematobium* eggs were found by microscopy in 15.5% of the samples with three previous negative tests, the true prevalence can be as high as 63.5% if the distribution for negative indirect tests is maintained. No statistical differences were found on the distribution of the infection by sex, age or previous treatment with praziquantel. Proximity to a source of water was associated with an increased likelihood of infection (Odds ratio 1.97 (1.45–2.70) *p* < 0.001). These data are shown in Table 1.

**Table 1:** Risk factors for schistosomiasis infection.

	Total screened children n: 1.425	Number of children infected by *S*.*haematobium* n: 785	OR (CI 95%)	p
**Gender**				
Girls	710	390/710 (54.9%)	1.08 (0.86–1.35)	0.55
**Age**				
= <5	81	49/81 (60.4%)	1	
6–8	434	248/434 (57.1%)	0.84 (0.50–1.40)	0.59
9–11	568	319/568 (56.1%)	0.64 (0.39–1.06)	0.11
>11	265	169/265 (63.7%)	1.05 (0.62–1.80)	0.94
Unknown	77			
**Distance to the source of water**				
>1500	219	108/219 (49.3%)	1	
1000-1500m	162	87/162 (53.7%)	1.19 (0.79–1.70)	0.45
500-1000m	158	102/158 (64.6%)	1.87 (1.22–2.84)	<0.05
<500m	742	488/742 (65.7%)	1.95 (1.43–2.64)	<0.001
Unknown	0			
**Previous treatment with Praziquantel**				
Treated	65	38/65 (58.2%)	0.89 (0.54–1.48)	0.75
Unknown	111			

OR: odds ratio. CI: confidence interval.

### Estimated global prevalence of schistosomiasis through indirect tests

#### Colorimetric test

We made a total of 1.283 colorimetric tests. Among these, 539 children (42%) reported haematuric urine (39.3–44.8, CI 95%).

#### Micro/macro haematuria

A total of 1,283 urine samples were observed for macroscopic and microscopic haematuria. The dipstick urine test showed one cross of haematuria in 189 samples (14.7%); two crosses on 131 (10.24%) and three crosses on 524 (40.96%). Therefore, 844 samples were positive for microhaematuria equating to an estimated prevalence of 65.7% (59.8–72.7, CI 95%). Macroscopic observation of haematuria was positive on 219 samples, equating to an estimated global prevalence of 17.06% (15.15–19.35, CI 95%).

Sensitivity, specificity, positive predictive value, false positives and negative and positive likelihood ratios of all indirect tests compared with the gold standard (direct visualization of *S*. *haematobium* eggs) are detailed in Table 2.

**Table 2:** Indirect tests utility compared to gold standard (urine parasitological examination).

	Positive tests/tests performed	Sensitivity (%)	Specificity (%)	Positive predictive value (%)	False positive (%)	Positive likelihood ratio	Negative likelihood ratio
**Urine dipstick**	844/1283	96.0	61.3	88.8	11.1	5.09	0.05
**Haematuria**	219/1283	27.1	97.5	97.2	0.2	22.63	0.74
**Colorimetric test**	539/1283	52.5	74.6	77.0	20.3	2.07	0.64
**Gold standard (Direct examination)**	785/1283						

Considering proteinuria, 1,283 tests were performed. Six hundred and fifty-three (51.1%) samples were positive, 244 (19.1%) had one cross, 220 (17.2%) two crosses and 189 (14.8%) three crosses. Proteinuria was found more frequently in the over-11 child age group (57.3%) and less frequently in the under-five group (33%). The more severe degree of infection (three crosses) was also more frequent in this group (21.5%). Also, it was much more frequent in the children that had *S*. *haematobium* eggs in urine (75.2%). The relationship between the degree of proteinuria with age and detection of *S*. *haematobium* eggs in urine samples is shown in Table 3.

**Table 3:** Relationship of proteinuria with other parameters.

Samples with proteinuria (n %)	Age					Eggs of *Schistosoma haematobium*		
	= <5 n = 39	6–8 n = 406	9–11 n = 537	> = 12 n = 260	P	Positive n = 785	Negative n = 498	P
+ (30 mg/dL)	5 (12.8%)	74 (16.7%)	105 (19.6%)	60 (23.1%)	<0.01	197 (25.2%)	47 (9.4%)	<0.01
++ (100 mg/dL)	6 (15.4%)	92 (20.8%)	89 (16.6%)	33 (12.7%)		209 (26.6%)	12 (2.4%)	
+++ (300 mg/dL)	2 (5.1%)	75 (16.9%)	56 (10.4%)	56 (21.5%)		186 (23.7%)	6 (1.2%)	
Positive	13 (33.3%)	241 (54.4%)	250 (46.6%)	149 (57.3%)		592 (75.4%)	65 (13.0%)	
Negative	26 (66.7%)	202 (45.6%)	287(53.4%)	111 (42.7%)		193 (24.5%)	433 (87.0%)	
**TOTAL**	**39 (100%)**	**406 (100%)**	**537 (100%)**	**260 (100%)**		**785 (100%)**	**498 (100%)**	

### Other parasites’ infection prevalence

One hundred and ninety-one stool samples were collected. None was positive for *S*. *mansoni*; Eight (4%) were positive for *S*. *haematobium*. The prevalence of various species of intestinal parasites was 16.7% (12.5–-23.7 CI 95%), as in Table 4.

**Table 4:** Prevalence of intestinal parasites.

Parasite	N	(%)	CI 95%
Hookworm	11	5.79	2.93–10.12
*Hymenolepis nana*	10	5.26	2.55–9.47
*Taenia sp*.	5	2.63	0.86–6.03
*Ascaris lumbricoides*	5	2.63	0.86–6.03
*Hymenolepis diminuta*	2	1.05	0.13–3.75
*Strongyloides stercoralis*	1	0.53	0.01–2.90

CI = Confidence Interval

## Discussion

The prevalence of urinary schistosomiasis in the studied population (61%) is very high, much higher than the national average (28%); these data imply that urinary schistosomiasis is a major public health problem in the area; according to the WHO guidelines [9] the population as a whole must be considered as a high-risk community and annual treatment campaigns should be initiated. Furthermore, these data should be used as a baseline for implementation of a wide range of measures that have been proven effective for schistosomiasis control, such as health education, clean water supply facilities, sanitation and ecological intervention when possible [10]. Further studies are needed in order to obtain data about related morbidity and mortality, or prevalence in other age groups. Cubal River surrounds the city of Cubal; throughout its course it forms a significant number of water bodies appropriate for bathing; there is also a pond in the north of the city where people wash their clothes. Once all the risk factors were evaluated, the proximity to one of these sources of water was the only one associated with urinary schistosomiasis. Proximity to a source of water where snail habitats flourish and people are exposed to the water correlates with areas of high human schistosomiasis prevalence [11,12]; this has also been shown to be the case in other endemic areas in northern Angola, mainly endemic for *S*. *haematobium* [5]. Although not surprising, to highlight the river and the pond as transmission areas for urinary schistosomiasis might provide a useful baseline for future malacological studies and ecological interventions, including detection of hot-spots where environmental intervention could have an impact. No differences of prevalence were found in relation to age or gender, even considering children younger than five years old (despite the low number of children in this group of age included in this analysis). These results reinforce the idea that transmission in the area is really high. This has been shown in areas highly endemic for intestinal and urinary schistosomiasis [13,14]. Further studies should be performed to assess the prevalence, disease burden, and safety and efficacy of treatment in pre-school-age children, as they are not usually included in official mass treatment campaigns.

Regarding the different indirect diagnostic tests, the most useful was the urine dipstick, with a sensitivity of 96% and a specificity of 61%; these results are similar to others reported in previous studies [15–17]. Because of its accuracy, ease of use and quick interpretation it can be considered as a reliable marker for the rapid diagnosis of urinary schistosomiasis in a low-resource, highly endemic context. The colorimetric test showed a lower performance, with a sensitivity of 52% and a specificity of 75%; nevertheless, its positive predictive value was 77%; despite its limitations, this test offers several advantages, mainly its ease of use, even by non-qualified staff, at a much lower cost, as no laboratory material or infrastructure is needed. Moreover, it can be conducted directly in the field, so treatment can be administered at the same time. This method has been previously reported [18, 19] and it is accepted as a useful tool in high-endemic areas where application of multifaceted approaches is needed in order to optimize resources. For such cases, we propose a combination of methods, in order to reach as many infected children as possible; if urine is haematuric, treatment could be directly administered; in campaigns in highly affected neighbourhoods, treatment based on the colorimetric test could be a reasonable approach, though cost-effectiveness studies should be conducted in the area. Urine dipstick testing, if available, is a reasonable approach to diagnosis in the absence of rapid access to microscopy.

Interestingly, 51% of the samples showed some degree of proteinuria; most of these cases presented concomitantly haematuria and *S*. *haematobium* eggs and the older children were more affected; the relationship of *S*. *haematobium* presence and proteinuria has been shown previously [20, 21], but further studies are required to determine whether the presence of proteinuria is associated with structural damage in the upper urinary tract, or a worse prognosis, even at such a young age.

Despite urinary schistosomiasis and macroscopic haematuria being common among children in the community, very few had received treatment with praziquantel (4.5% in the previous two months), showing the lack of preventive chemotherapy use in the area [6]. Further studies of the knowledge and beliefs about the disease among community and health workers could help to guide future educational campaigns [22, 23].

We did not detect the presence of intestinal schistosomiasis in the study area; this finding is consistent with the clinical impression and with previous reports [5, 7], given that both species of schistosomiasis are present in different regions in Angola, showing a patchy distribution. This is possibly due to geographical and ecological factors, but it should be confirmed with malacological studies which could identify the specific species of fresh water snails present in the area.

Although other parasitic infections were not rare (16.75%) in the studied population, prevalence was lower than the national average (40% in school-aged children [6]), mainly in the case of *Strongyloides stercoralis*. This lower ratio could be explained by diverse factors. We do not believe that previous treatment could be the reason, as geohelminths mass treatment campaigns have not been implemented on a regular basis in this area and only 1.5% children had taken any antihelmintic treatment in the two previous months; the difficulty in collecting faecal samples, which conditioned a low number of specimens, and the processing technique used (formol-ether) in only one sample are likely reasons for the underestimation of the prevalence of geohelminths, mainly for *Strongyloides stercoralis* [24], as larvae of this parasite are better visualized on direct examination. These are the main limitations of this study. A secondary survey focused on intestinal helminths is warranted, in order to assess more precisely the prevalence and intensity levels of infection.

A not negligible amount of *S*. *haematobium* eggs in faeces was found. This is probably the result of a hyperparasitation that leads to migration of parasites to the intestinal vessels; this phenomenon can be explained by a spill-over caused by high loads of parasites [25]. Another possible explanation, however less likely, might be contamination of faeces with urine, mainly in the case of girls or very small children; however, this is not supported by the study’s data, as 6/8 (75%) children with *S*. *haematobium* eggs in faeces were boys and 8/8 (100%) were eight or more years old. An explanation that has been suggested in other studies is sexual interaction between the two species of schistosomes (*S*. *haematobium* and *S*. *mansoni*) [26, 27], but this is unlikely in this study, as no *S*. *mansoni* was diagnosed. More studies are required to determine whether different species of snails than previously described could have a role in these cases.

This survey is one of the few studies conducted in Angola into the epidemiology of neglected tropical diseases, particularly schistosomiasis. It is the result of a coordinated effort between educational authorities and the local administration and this is one of its mains strengths, as it allowed for successful planning and implementation of the study, as well as a good way to approach the community facilitating educational tasks. More importantly, such collaboration will be essential for future interventions that are warranted in the community, such as implementation of preventive therapy, ecological studies or educational tasks. Additional information, such as socio-economic characteristics, life habits in relation to the use of water and latrines should be included in further surveys.

### Conclusions

The prevalence of urinary schistosomiasis in the school-age population of Cubal, Angola, is very high, indicating the need for urgent intervention at a public health level, the major risk factor being the proximity to one of the main sources of water. Indirect tests, such as urine dipstick and colorimetric tests are useful tools for diagnosis in this context. Further studies are needed in order to assess more accurately the prevalence of intestinal schistosomiasis and geohelminths.

## Supporting Information

S1 Checklist STROBE checklist. (DOCX)

## References

[1] King CH, Dickman K, Tisch DJ. Reassessment of the cost of chronic helmintic infection: a meta-analysis of disability-related outcomes in endemic schistosomiasis. Lancet 2005;365:1561–9. 15866310 10.1016/S0140-6736(05)66457-4

[2] Lozano R, Naghavi M, Foreman K, Lim S, Shibuya K, Aboyans V, et al Global and regional mortality from 235 causes of death for 20 age groups in 1990 and 2010: a systematic analysis for the Global Burden of Disease Study 2010. Lancet 2012;380:2095–128. 10.1016/S0140-6736(12)61728-0 23245604 PMC10790329

[3] Tohon ZB, Mainassara HB, Garba A, Mahamane AE, Bosqué-Oliva E, Ibrahim M-L, et al Controlling schistosomiasis: significant decrease of anaemia prevalence one year after a single dose of praziquantel in Nigerian schoolchildren. PLoS Negl Trop Dis 2008;2:e241 10.1371/journal.pntd.0000241 18509472 PMC2386241

[4] WHO (2002) Prevention and Control of Schistosomiasis and Soil-Transmitted Helminthiasis Report of a WHO Expert Committee. Geneva: World Health Organization. 12592987

[5] Sousa-Figueiredo JC, Gamboa D, Pedro JM, Fançony C, Langa AJ, Magalhães RJS, et al Epidemiology of malaria, schistosomiasis, geohelminths, anemia and malnutrition in the context of a demographic surveillance system in northern Angola. PLoS ONE 2012;7:e33189 10.1371/journal.pone.0033189 22493664 PMC3320883

[6] MINSA, WFP, WHO, UNICEF (2005) Baseline survey for helminth control in school-aged children in Angola Luanda: Ministerio de Saude

[7] - Atlas of the global distribution of schistosomiasis. CEGET-CNRS/ OMS-WHO 1987.

[8] - Perfil do Município do CUBAL, Província de Benguela 2009. Administraçao Municipal do Cubal, Ediçoes de Angola Lda (EAL), Octubre 2009.

[9] Montresor A., Crompton D.W.T., Hall A:, Bundy D.A.P. and Savioli L. Guidelines for the evaluation of soil-transmitted helminthiasis and schistosomiasis at community level. WHO/CTD/SIP/98.1

[10] Lee Y-H, Jeong HG, Kong WH, Lee S-H, Cho H-I, Nam H-S, et al Reduction of urogenital schistosomiasis with an integrated control project in Sudan. PLoS Negl Trop Dis 2015;9:e3423 10.1371/journal.pntd.0003423 25569278 PMC4288734

[11] Opisa S, Odiere MR, Jura WGZO, Karanja DMS, Mwinzi PNM. Malacological survey and geographical distribution of vector snails for schistosomiasis within informal settlements of Kisumu City, western Kenya. Parasit Vectors 2011;4:226 10.1186/1756-3305-4-226 22152486 PMC3247081

[12] Woodhall DM, Wiegand RE, Wellman M, Matey E, Abudho B, Karanja DMS, et al Use of Geospatial Modeling to Predict Schistosoma mansoni Prevalence in Nyanza Province, Kenya. PLoS One 2013;8. 10.1371/journal.pone.0071635 PMC3743764 23977096

[13] Betson M, Sousa-Figueiredo JC, Kabatereine NB, Stothard JR. New Insights into the Molecular Epidemiology and Population Genetics of Schistosoma mansoni in Ugandan Pre-school Children and Mothers. PLoS Negl Trop Dis 2013;7. 10.1371/journal.pntd.0002561 PMC3861247 24349589

[14] Coulibaly JT, N’Gbesso YK, N’Guessan NA, Winkler MS, Utzinger J, N’Goran EK. Epidemiology of schistosomiasis in two high-risk communities of south Cote d’Ivoire with particular emphasis on pre-school-aged children. Am J Trop Med Hyg 2013;89:32–41. 10.4269/ajtmh.12-0346 23690549 PMC3748484

[15] Lwambo NJS, Savioli L, Kisumku UM, Alawi KS, Bundy DAP (1997). Control of Schistosoma haematobium morbidity on Pemba Island: validity and efficiency of indirect screening tests. Bull World Health Organ 75: 247–252. 9277012 PMC2486950

[16] Mafe MA. The diagnostic potential of three indirect tests for urinary schistosomiasis in Nigeria. Acta Trop 1997;68:277–84. 9492912 10.1016/s0001-706x(97)00102-2

[17] King CH, Bertsch D. Meta-analysis of urine heme dipstick diagnosis of Schistosoma haematobium infection, including low-prevalence and previously-treated populations. PLoS Negl Trop Dis 2013;7:e2431 10.1371/journal.pntd.0002431 24069486 PMC3772022

[18] Lengeler C, Utzinger J & Tanner M (2002) Questionnaires for rapid screening of schistosomiasis in sub-Saharan Africa. Bulletin of the World Health Organization 80, 235–242 11984610 PMC2567742

[19] Kihara J, Mwandawiro C, Waweru B, Gitonga CW, Brooker S. Preparing for national school-based deworming in Kenya: the validation and large-scale distribution of school questionnaires with urinary schistosomiasis. Tropical Medicine and International Health. Volume 16 no 10 pp 1326–1333 10 2011 10.1111/j.1365-3156.2011.02829.x 21767334 PMC3558801

[20] Wami WM, Nausch N, Midzi N, Gwisai R, Mduluza T, Woolhouse M, et al Identifying and evaluating field indicators of urogenital schistosomiasis-related morbidity in preschool-aged children. PLoS Negl Trop Dis 2015;9:e0003649 10.1371/journal.pntd.0003649 25793584 PMC4368198

[21] Brouwer KC, Ndhlovu PD, Wagatsuma Y, Munatsi A, Shiff CJ. Epidemiological assessment of Schistosoma haematobium-induced kidney and bladder pathology in rural Zimbabwe. Acta Trop 2003;85:339–47. 12659971 10.1016/s0001-706x(02)00262-0

[22] Omedo M, Ogutu M, Awiti A, Musuva R, Muchiri G, Montgomery SP, et al The effect of a health communication campaign on compliance with mass drug administration for schistosomiasis control in western Kenya—the SCORE project. Am J Trop Med Hyg 2014;91:982–8. 10.4269/ajtmh.14-0136 25246690 PMC4228896

[23] Yuan L-P, Manderson L, Ren M-Y, Li G-P, Yu D-B, Fang J-C. School-based interventions to enhance knowledge and improve case management of schistosomiasis: a case study from Hunan, China. Acta Trop 2005;96:248–54. 16202594 10.1016/j.actatropica.2005.07.019

[24] Krolewiecki AJ, Lammie P, Jacobson J, Gabrielli A-F, Levecke B, Socias E, et al A public health response against Strongyloides stercoralis: time to look at soil-transmitted helminthiasis in full. PLoS Negl Trop Dis 2013;7:e2165 10.1371/journal.pntd.0002165 23675541 PMC3649958

[25] Husting EL. Comments on “the routes of schistosome egg passage…” Cent Afr J Med 1965;11:250–4. 5833090

[26] Cunin P, Tchuem Tchuenté L-A, Poste B, Djibrilla K, Martin PMV. Interactions between Schistosoma haematobium and Schistosoma mansoni in humans in north Cameroon. Trop Med Int Health 2003;8:1110–7. 14641846 10.1046/j.1360-2276.2003.01139.x

[27] Webster BL, Southgate VR, Tchuem Tchuenté LA. Mating interactions between Schistosoma haematobium and S. mansoni. J Helminthol 1999;73:351–6. 10654406

