# Supplementary material for: Epidemiology of Schistosomiasis and Usefulness of Indirect Diagnostic Tests in School-Age Children in Cubal, Central Angola
Source: PLoS Negl Trop Dis. 2015 Oct 16;9(10):e0004055. doi: 10.1371/journal.pntd.0004055 (PMC4608768; doi:10.1371/journal.pntd.0004055)
Supplement: S1 Checklist — (DOCX) [file pntd.0004055.s001.docx]

STROBE Statement—Checklist of items that should be included in reports of ***cross-sectional studies***

|  | Item No | Recommendation |
| --- | --- | --- |
| **Title and abstract** | 1 | (*a*) Indicate the study’s design with a commonly used term in the title or the abstract. |
|  |  | (*b*) Provide in the abstract an informative and balanced summary of what was done and what was found  **We have included this information in the abstract** |
| Introduction | | |
| Background/rationale | 2 | Explain the scientific background and rationale for the investigation being reported  **We have explained the scientific background in the introduction** |
| Objectives | 3 | State specific objectives, including any prespecified hypotheses  **We have specified our objectives** |
| Methods | | |
| Study design | 4 | Present key elements of study design early in the paper  **We have explained our study design in the methods section** |
| Setting | 5 | Describe the setting, locations, and relevant dates, including periods of recruitment, exposure, follow-up, and data collection  **We have described setting and relevant dates** |
| Participants | 6 | Give the eligibility criteria, and the sources and methods of selection of participants  **We have described the eligibility criteria and the methods of selection** |
| Variables | 7 | Clearly define all outcomes, exposures, predictors, potential confounders, and effect modifiers. Give diagnostic criteria, if applicable  **We have described all the factors risk, exposures and outcomes in the methods section. We have described all the used techniques** |
| Data sources/ measurement | 8* | For each variable of interest, give sources of data and details of methods of assessment (measurement). Describe comparability of assessment methods if there is more than one group  **We have included and compared the different diagnostic procedures** |
| Bias | 9 | Describe any efforts to address potential sources of bias  **We have described our potential limitations and our methodology in order to avoid bias** |
| Study size | 10 | Explain how the study size was arrived at  **We have explained our methodology to calculate the study size** |
| Quantitative variables | 11 | Explain how quantitative variables were handled in the analyses. If applicable, describe which groupings were chosen and why  **We have explained our methodology about quantitative variables** |
| Statistical methods | 12 | (*a*) Describe all statistical methods, including those used to control for confounding |
|  |  | (*b*) Describe any methods used to examine subgroups and interactions |
|  |  | (*c*) Explain how missing data were addressed |
|  |  | (*d*) If applicable, describe analytical methods taking account of sampling strategy |
|  |  | (*e*) Describe any sensitivity analyses  **We have explained this information about statistical methods** |
| Results | | |
| Participants | 13* | (a) Report numbers of individuals at each stage of study—eg numbers potentially eligible, examined for eligibility, confirmed eligible, included in the study, completing follow-up, and analysed |
|  |  | (b) Give reasons for non-participation at each stage |
|  |  | (c) Consider use of a flow diagram  **We have included information about participants** |
| Descriptive data | 14* | (a) Give characteristics of study participants (eg demographic, clinical, social) and information on exposures and potential confounders |
|  |  | (b) Indicate number of participants with missing data for each variable of interest  **We have explained characteristics of study participants** |
| Outcome data | 15* | Report numbers of outcome events or summary measures  **We have explained all possible outcomes and different ways of diagnosis and we have compared them** |
| Main results | 16 | (*a*) Give unadjusted estimates and, if applicable, confounder-adjusted estimates and their precision (eg, 95% confidence interval). Make clear which confounders were adjusted for and why they were included |
|  |  | (*b*) Report category boundaries when continuous variables were categorized |
|  |  | (*c*) If relevant, consider translating estimates of relative risk into absolute risk for a meaningful time period  **We have explained all our main results in terms of risk and precision, we have reported category boundaries of continuous variables** |
| Other analyses | 17 | Report other analyses done—eg analyses of subgroups and interactions, and sensitivity analyses  **We have made sensitivity, specificity and predictive values measures to compare different diagnostic tests** |
| Discussion | | |
| Key results | 18 | Summarise key results with reference to study objectives |
| Limitations | 19 | Discuss limitations of the study, taking into account sources of potential bias or imprecision. Discuss both direction and magnitude of any potential bias |
| Interpretation | 20 | Give a cautious overall interpretation of results considering objectives, limitations, multiplicity of analyses, results from similar studies, and other relevant evidence |
| Generalisability | 21 | Discuss the generalisability (external validity) of the study results  **We have summarized key results, discussed our main limitations and considered interpretation of our findings** |
| Other information | | |
| Funding | 22 | Give the source of funding and the role of the funders for the present study and, if applicable, for the original study on which the present article is based  **We have discussed about our funding** |

*Give information separately for exposed and unexposed groups.
